# Distinct Mutations Led to Inactivation of Type 1 Fimbriae Expression in *Shigella* spp.

**DOI:** 10.1371/journal.pone.0121785

**Published:** 2015-03-26

**Authors:** Verónica Bravo, Andrea Puhar, Philippe Sansonetti, Claude Parsot, Cecilia S. Toro

**Affiliations:** 1 Programa de Microbiología, ICBM, Facultad de Medicina, Universidad de Chile, Santiago, Chile; 2 Unité de Pathogénie Microbienne Moléculaire, Institut Pasteur, Paris, France; 3 INSERM, Paris, France; New York State Dept. Health, UNITED STATES

## Abstract

*Shigella* spp. are responsible for bacillary dysentery in humans. The acquisition or the modification of the virulence plasmid encoding factors promoting entry of bacteria into and dissemination within epithelial cells was a critical step in the evolution of these bacteria from their *Escherichia coli* ancestor(s). Incorporation of genomic islands (GI) and gene inactivation also shaped interactions between these pathogens and their human host. Sequence analysis of the GI inserted next to the *leuX* tRNA gene in *S*. *boydii*, *S*. *dysenteriae*, *S*. *flexneri*, *S*. *sonnei* and enteroinvasive *E*. *coli* (EIEC) suggests that this region initially carried the *fec*, *yjhATS* and *fim* gene clusters. The *fim* cluster encoding type I fimbriae is systematically inactivated in both reference strains and clinical isolates and distinct mutations are responsible for this inactivation in at least three phylogenetic groups. To investigate consequences of the presence of fimbriae on the outcome of the interaction of *Shigella* with host cells, we used a *S*. *flexneri* strain harboring a plasmid encoding the *E*. *coli fim* operon. Production of fimbriae by this recombinant strain increased the ability of bacteria to adhere to and enter into epithelial cells and had no effect on their ability to disseminate from cell to cell. The observations that production of type I fimbriae increases invasion of epithelial cells and that independent mutations abolish fimbriae production in *Shigella* suggest that these mutations correspond to pathoadaptive events.

## Introduction

Members of *Shigella* spp. and enteroinvasive *Escherichia coli* (EIEC) are responsible of bacillary dysentery, a major cause of diarrheal diseases in humans [1], [2]. Bacteria invading, multiplying and disseminating in the colonic epithelium induce an acute inflammation of the colon. The Mxi-Spa type III secretion (T3S) system promoting entry of bacteria into epithelial cells and the outer membrane protein IcsA promoting the actin-based motility of intracellular bacteria and their dissemination from cell to cell are encoded by a 220-kb virulence plasmid [3], [4]. The acquisition of the virulence plasmid, or its construction from elements of various origins, was a critical step in the evolution of *Shigella* spp. from their *E*. *coli* ancestor(s). Further, incorporations of genomic islands (GI) encoding such pathogenicity factors as the Shiga toxin of *S*. *dysenteriae* [5], the aerobactin transport system [6] and the SigA and PicA proteases [7] also shaped the interactions between *Shigella* and its host [8], [9].

Among *Shigella* and EIEC strains, population genetic studies identified six phylogenetic groups (S1, S2, S3, SS, SD1 and A) interspersed within *E*. *coli* phylogenetic groups (A, B1, B2, D and E) [10]–[12]. As compared to *E*. *coli*, *Shigella* contains a large number of inactivated genes [13], [14]. These genes might have been inactivated either because they were no longer useful to bacteria following the acquisition of the ability to invade the mucosa, or because their expression was detrimental to the multiplication and survival of bacteria in this new environment [15]. The detection of distinct mutations leading to the inactivation of the same gene (or pathway) in different lineages is suggestive of pathoadaptive mutations [16], [17]. In addition, the analysis of strains in which expression of a gene of interest is experimentally reactivated can provide evidence in support of pathoadaptive mutations. For example, the *cad* cluster involved in production of cadaverine and the *nadA* and *nadB* genes involved in the synthesis of quinolinate are inactivated in *Shigella* spp. and experimental restoration of the production of these compounds attenuated the virulence of recombinant strains [18]–[20].

Type 1 fimbriae are filamentous surface structures produced by several members of the Enterobacteriaceae family [21]. These fimbriae are encoded by the *fimAICDFGH* operon containing genes required for their assembly and structure [22]–[24]. FimA is the major structural subunit of fimbriae, FimI is required for fimbriae biosynthesis although its exact role is not known, FimC is the periplasmic chaperone for fimbriae subunits, FimD is the outer-membrane assembly platform, FimF and FimG are adaptor proteins and FimH is the adhesin located at the tip of fimbriae and mediating adhesion of bacteria to mannose containing molecules on host mucosal surfaces [25]–[28]. Phase-variable expression of fimbriae is mediated by the inversion of the 314-bp invertible element *fimS* containing the promoter of the *fim* operon; inversion is controlled by the two site-specific tyrosine recombinases FimE and FimB encoded by genes located upstream from *fimS* [29], [30]. FimB promotes inversion of *fimS* in both directions, while FimE catalyzes the ON-to-OFF inversion. The *fim* gene cluster is present within the GI adjacent to the tRNA *leuX* gene in *E*. *coli*.

In the present work, we investigated the role of type I fimbriae in the invasive phenotype of *Shigella* spp. Sequence analysis of published genomes and whole genome shotgun (WGS) data of representative members of *Shigella* spp. and EIEC strains and PCR analysis of *S*. *flexneri* clinical isolates indicated that the *fim* cluster is inactivated in all strains and that different mutations are responsible for its inactivation in at least three phylogenetic groups. To analyze the behavior of a fimbriated *S*. *flexneri* strain in *in vitro* models of infection, we used a recombinant *S*. *flexneri* strain harboring a plasmid encoding the *E*. *coli fim* cluster. Production of fimbriae led to a 50-fold increase in the ability of bacteria to adhere to and invade epithelial cells. The fact that these pathogens lost fimbriae expression in spite of the superior capacity to invade host cells conferred by fimbriae suggests that inactivation of fimbriae production represents a new pathoadaptive event in *Shigella* spp.

## Materials and Methods

### Bacterial strains, plasmids and media

The *S*. *flexneri* strain M90T (serotype 5a) was used as the wild-type reference strain [31]. The strain BS176 is a noninvasive derivative of M90T cured of the virulence plasmid pWR100 [31], [32]. The plasmid pSH2 harboring the *fim* operon from the uropathogenic *E*. *coli* strain J96 [22] is a derivative of the vector pACYC184. The *E*. *coli fim* operon exhibits 97% sequence identity with the *fim* operon present at the *leuX* locus of the *S*. *flexneri* strain 2457T. Clinical isolates were collected from stool samples of Chilean children aged <14 years with acute diarrhea between 2004 to 2006 [33]. *S*. *flexneri* reference and clinical strains, as well as *S*. *sonnei* strains, were grown in trypticase soy broth medium (TCS) containing chloramphenicol (25 μg ml^-1^) when appropriate. To enrich the population of bacteria expressing fimbriae, bacteria were first passaged twice in liquid medium under static conditions at 37°C for 24 h. Then, to screen for bacteria still harboring the virulence plasmid, bacteria were isolated on agar plates containing Congo red. Bacteria from red colonies were passaged again in liquid medium under static conditions for 24 h, sub-cultured at 37°C for 3–4 h with agitation and harvested by centrifugation (3,000 x g for 10 min at 4°C). For adhesion and invasion assays, bacteria were washed with PBS and suspended in Dulbecco Modified Earl’s Medium (DMEM) containing glucose (1 g l^-1^) and HEPES, pH 7.4, (20 mM). The orientation of the *fim* promoter in bacteria used for infections was monitored by restriction analysis of a PCR product covering *fimS* (see below).

### Genome and WGS accession numbers

Strains for which genomes sequences and WGS results were used in this study are the following: *E*. *coli* MG1655, NC_000913.3 [34]; EIEC 53638 (serotype O144), AAKB02000001.1; *S*. *boydii* 4444–74 (serotype 2), NZ_AKNB1000000; *S*. *boydii* Sb227 (serotype 4), CP000036 [35]; *S*. *boydii* 3594–74 (serotype 4), NZ_AFGC000000.1; *S*. *boydii* strains CIP52-54 (serotype 7), ERR200284 (F.-X. Weill, personal communication); *S*. *boydii* CIP56-18 (serotype 11), ERR200299 (F.-X. Weill, personal communication); *S*. *boydii* CDC3083-94 (serotype 18), NC_010658; *S*. *dysenteriae* Sd197 (serotype 1), CP000034 [35]; *S*. *dysenteriae* 155–74 (serotype 2), NZ_AFFZ01000000; *S*. *flexneri* 2457T (serotype 2a), AE014073 [13]; *S*. *flexneri* CP301 (serotype 2a), AE005674 [36]; *S*. *flexneri* NCTC1 (serotype 2a), LM651928 [37]; *S*. *flexneri* M90T (serotype 5a), CM001474.1 [38]; *S*. *flexneri* Sf8401 (serotype 5b), CP000266 [39]; *S*. *flexneri* CFSAN027317 (ATCC 12025) (serotype 6), JWSL01000004.1; *S*. *flexneri* CCH60 (serotype 6), AKMW01000080.1; *S*. *flexneri* CDC 796–83 (serotype 6), AERO01000069.1; *S*. *flexneri* collection of 59 strains covering 14 serotypes (1a, 1b, 1d, 2a, 2b, 3a, 3b, 4a, 4av, 4b, X, Xv, Y and Yv), AZOG00000000.1 to AZQM00000000.1 [40]; *S*. *flexneri* collection of the 16 type strains covering all the established serotypes (1a, 1b, 1c, 2a, 2b, 3a, 3b, 3c, 4a, 4b, 5a, 5b, 6, X, Y and E1037), ERS088060-ERS088076 [41]; *S*. *sonnei* Ss046, CP000038 [35]; *S*. *sonnei* strain 53G, HE616528.

### Characterization of the *leuX* GI in clinical isolates

The presence and the organization of the GI at the *leuX* locus were characterized by tiling-PCR and restriction analysis. The genomic DNA was purified using the Genomic DNA Purification Kit from Promega®. Primers (Table 1) used to amplify 6 fragments covering the whole GI were designed according to the sequence of the *S*. *flexneri* 2a strains 2457T. PCR fragment 3 containing *fimB* was analyzed by using the restriction enzyme *Bfa*I (Fermentas). Sizes of *Bfa*I fragments obtained from most strains were 1862, 1583 and 258 bp; the presence of the 258-bp fragment indicates the presence of a mutation at codon 162 of *fimB*. In strain 2457T, the 1583-bp fragment was replaced by 1392- and 191-bp fragments, due to the presence of an additional *Bfa*I site at codon 130 of *fimA*.

**Table 1:** Primers used for the characterization of the GI *leuX*.

Primer Name	Primer sequence (5'-3')
**Inv-1**	CAGTAATGCTGCTCGTTTTGCCG
**Inv-2**	GACAGAGCCGACAGAACAACG
**Frag 1-F**	TGGTGACGATCCCAAGTGTA
**Frag 1-R**	CCTGTGGTAATGCCGTTTCT
**Frag 2-F**	AGAAGCTGTATTCCCAGTCC
**Frag 2-R**	TAACCAATTGCCACAGGACC
**Frag 3-F**	GTTCCGGCATTCAACTCTGT
**Frag 3-R**	AACAACGCACCCGCTATTGA
**Frag 4-F**	CGAATAGCGTAACATGTGCG
**Frag 4-R**	ACGCGTAGTCACTGGTCATT
**Frag 5-F**	ATATTCAGAACGGCACGGAG
**Frag 5-R**	ACTATTGGTCTGGTGCTGGT
**Frag 6-F**	TACCTGCTAAACCAGTACCC
**Frag 6-R**	ACCTTGCTCGCAGTTGATCT

### Determination of the orientation of the *fim* promoter region

The orientation of the invertible DNA element *fimS* carrying the *fim* promoter was determined as previously described [42]. Briefly, primers Inv-1 and Inv-2 (hybridizing to each side of *fimS*) were used to amplify a 601-bp DNA fragment that was digested with *Sna*BI (Fermentas) and restriction fragments were resolved on 2% agarose gels. Due to the asymmetric location of the *Sna*BI cleavage site within the invertible element, different restriction fragments were obtained depending on the element orientation: the phase ON orientation yielded fragments of 403 and 198 bp whereas the phase OFF orientation yielded fragments of 440 and 161 bp.

### Electron Microscopy

To visualize type 1 fimbriae on the bacterial surface, a drop of bacterial suspensions was placed onto a cupper electron microscope grid for 15 minutes at 20°C. The suspension was removed by absorption with a filter paper and the sample was fixed with a solution containing 5% formaldehyde and stained with 0.5% uranyl acetate prior to examination. Samples were analyzed on Jeol 1200 EX set at 80 kV.

### Haemagglutination assay

The presence of functional type 1 fimbriae on the bacterial surface was tested using a modified protocol of the mannose-sensitive haemagglutination assay (MSHA) on Guinea pig erythrocytes [43], [44]. Briefly, equal volumes of a suspension containing 2×10^9^ bacteria per ml in PBS and a suspension of 5% Guinea pigs erythrocytes were mixed in the absence or in the presence of 0.2 mM mannose and agglutination was observed after 20 min of gentle shaking at 20°C.

### Adhesion, invasion and dissemination assays

HeLa and TC7 cells were grown in a humidified incubator at 37°C with 10% CO_2_ in DMEM (Hyclone®) supplemented with 10% fetal calf serum (FCS) (Hyclone®) and non-essential amino acids. HeLa cells (2x10^5^ cells) were grown in 12-well culture plates overnight to obtain a semi-confluent monolayer. For adhesion assays, HeLa cells were infected at a multiplicity of infection (MOI) of 100 bacteria per cell and plates were centrifuged at 180 x g for 10 min and incubated at 37°C for 30 min in the presence or in the absence of 0.2 mM mannose. Infected cells were washed five times with PBS and lysed in PBS containing sodium deoxycholate (0.1%, w/v). Cell-associated bacteria, both adherent onto the cell surface and intracellular, were quantified by plating dilutions of lysates onto LB agar plates. Data are the means and SD of three independent experiments performed in duplicate. In parallel, infected cells were fixed with ethanol and stained with Giemsa. The ability of bacteria to enter and disseminate within a differentiated monolayer of intestinal epithelial cells was evaluated using the plaque assay [45]. Briefly, TC7 cells were seeded into six-well plates and incubated at 37°C for 72 hours. Cells were infected at different MOIs (0.1, 0.01, 0.001 and 0.0001) in the absence or in the of presence 0.2 mM mannose and incubated for 2 h at 37°C without any centrifugation step. Infected cells were then washed, overlaid with DMEM containing agarose (0.5%, w/v), calf serum (10%, v/v) and gentamicin (50 μg ml^-1^) and incubated for 48 or 72 h at 37°C in the presence of CO_2._ The agarose layer was then removed and cells were fixed and stained with Giemsa.

### Statistical analysis

Data was analyzed either by one-way ANOVA or t-test. Statistical significance was assumed at a P-value ≤ 0.05. The data analysis was performed using the Prism 5.01 software (GraphPad).

## Results

### Analysis of the GI-*leuX* in *Shigella* spp. strains

**Fig 1:**
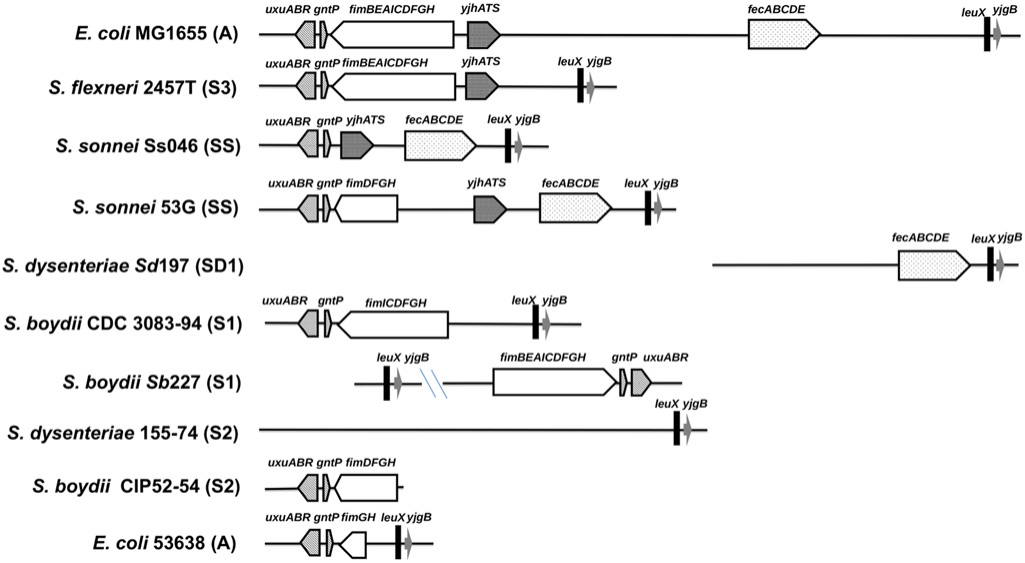
Genomic organization of the GI *leuX* in *E*. *coli* K12 and representative members of *Shigella* spp. A schematic representation (drawn to scale) of the main gene clusters present in the GI *leuX* is shown for the *E*. *coli* K-12 strain MG1655, the *S*. *flexneri* strain 2457T, the *S*. *sonnei* strain Ss046, the *S*. *dysenteriae* strain Sd197, the *S*. *boydii* strains CDC 3083–94 and Sb227, the *S*. *dysenteriae* strain 155–74, the *S*. *boydii* strain CIP52-54 and the EIEC strain 53638; the phylogenetic group to which each strain belongs is indicated in parenthesis. The GI encompasses the region located between *uxuABR-gntP* and *leuX*-*yjgB*. For the sake of clarity, IS (including IS inserted in *fim* genes) and phage remnants are not indicated.

In the *E*. *coli* K-12 strain MG1655, the *fim* cluster is located in a 50-kb GI also carrying the *fecABCDE* operon (encoding an iron transport system), the *yjhATS* genes (encoding factors involved in uptake and catabolism of sialic acids) and a number of IS elements and phage remnants. This GI is flanked by *uxuABR* (encoding proteins involved in hexuronate degradation) and *gntP* (encoding a fructuronate transporter) on one side and by *leuX* (encoding a Leu tRNA) and *yjgB* (encoding a predicted alcohol dehydrogenase) on the other side (Fig. 1). Comparison of the sequences of this region in MG1655 and representative strains of *S*. *flexneri*, *S*. *boydii*, *S*. *dysenteriae*, *S*. *sonnei* and EIEC from the six phylogenetic groups identified GIs of different sizes carrying various assortments of the *fim*, *yjh* and *fec* genes (Fig. 1); (i) group S3: the ~23-kb GI of *S*. *flexneri* strains 2457T and Sf301 (serotype 2a) and M90T and Sf8401 (serotypes 5a and 5b, respectively) carries the *fim* cluster and *yjhATS*; (ii) group SS: the 21-kb GI of the *S*. *sonnei* strain Ss046 carries *yjhATS* and *fec* genes; the 29-kb GI of the *S*. *sonnei* strain 53G carries the 3' part of the *fim* cluster, *yjhATS* and *fec* genes; (iii) group SD1: only *fec* genes are present in the vicinity of *leuX* and *yjgB* in *S*. *dysenteriae* strain Sd197 (serotype 1); the left boundary of the GI cannot be defined because the flanking *gntP* and *uxuABR* genes are missing; (iv) group S1: the 18-kb GI of the *S*. *boydii* strain CDC3083-94 (serotype 18) carries only a remnant of the *fim* cluster; in the *S*. *boydii* strain Sb227 (serotype 4), there is an inversion of a region encompassing part of the GI and only the *fim* cluster is present between *gntP* and *leuX*. In the absence of complete genome sequence data, we used WGS results for strains of the other phylogenetic groups; (v) group S2: in the *S*. *dysenteriae* strain 155–74 (serotype 2), the *fim*, *yjhATS* and *fec* genes, as well as the *uxuABR* and *gntP* genes, are absent; in *S*. *boydii* strains CIP52-54 and CIP56-18 of serotypes 7 and 11, respectively (F.-X. Weill, personal communication), only the 3' part of *fimD* and the *fimFGH* genes are present next to *gntP*; (vi) group A: in the EIEC strain 53638 (serotype O144), the 4-kb region located between *gntP* and *leuX* carries only the 3’ part of the *fim* cluster. These observations suggest that the *leuX* GIs harbored by *Shigella* and EIEC strains derive from a region carrying the *fec*, *yjhATS* and *fim* clusters and that different segments of this GI were lost or modified during the evolution of different phylogenetic groups.

Further analysis of the published sequences of the *fim* cluster revealed that *fimD* is interrupted by an IS*1* at codon 221 in the two *S*. *flexneri* strains of serotype 2a (2457T and Sf301) and *fimI* is interrupted by an IS*1* at codon 35 in the *S*. *flexneri* strains of serotypes 5a (M90T) and 5b (Sf8401). Furthermore, *fimB* is inactivated by a nonsense mutation at codon 162 (TAG instead of CAG in *E*. *coli* K12) in these four *S*. *flexneri* strains. In the *S*. *sonnei* strain 53G, the 3' end of *fimB* (from codon 188) up to the *fimC*-*fimD* intergenic region is replaced by an IS*1*, there is an insertion of IS*1* at codon 204 of *fimD* and of an unknown IS at codon 596 of *fimD*. In the *S*. *boydii* strain CDC3083-94, *fimB*, *fimE* and *fimA* are missing, *fimD* carries a frameshift mutation due to the deletion of one nucleotide at codon 27 and a nonsense mutation at codon 563 (TAG instead of CAG in *E*. *coli* K12) and an IS*1* is inserted at codon 98 of *fimC*. In the *S*. *boydii* strain Sb227 (serotype 4), *fimD* carries the same nonsense mutation at codon 563 and *fimH* is interrupted by an IS*629* at codon 264. There is an insertion of an IS*Sfl7* at codon 801 of *fimD* in the *S*. *boydii* strain CIP52-54 (serotype 7) and an insertion of an IS*600* at codon 509 of *fimD* in the *S*. *boydii* strain CIP56-18 (serotype 11); furthermore, the *fimBEAIC* genes are absent from these two strains. In the EIEC strain 53638, only the last two genes of the *fim* operon, *fimG* and *fimH*, are present. These observations indicate that the *fim* cluster is inactivated in *Shigella* and EIEC representative strains belonging to all phylogenetic groups.

### The *fim* operon is inactivated in *S*. *flexneri* clinical isolates

**Fig 2:**
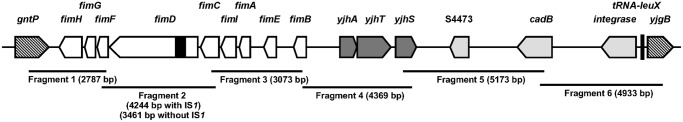
Characterization of the GI *leuX* in *S*. *flexneri*. The genetic organization of the ~23-kb GI inserted at the *leuX* locus in the *S*. *flexneri* reference strain 2457T is shown (drawn to scale). The *gntP* and *leuX*-*yjgB* genes flanking the GI are indicated by hatched arrows, *fim* genes by white arrows, *yjhATS* genes by dark-gray arrows and other genes present in the GI by pale-gray arrows. The position of IS*1* in *fimD* is indicated by a black box. The position and sizes of the six PCR fragments generated for the tiling PCR are indicated below the genetic map; the expected sizes of PCR fragment 2 corresponding to *fimD* with and without IS*1* are indicated. For the sake of clarity, other IS elements and phage remnants are not indicated.

To determine if the presence of the *fim* cluster is a common feature of *S*. *flexneri* strains and whether or not this gene cluster is complete and functional in clinical isolates, we analyzed a collection of 60 strains isolated from patients suffering from shigellosis in Chile between 2004 and 2006 [33]. Tiling-PCR was used to characterize the GI-*leuX* region in *S*. *flexneri* clinical isolates of serotypes 1a, 2a, 2b, 3a and 3b (Table 2), as well as in the reference strains 2457T (serotype 2a) and M90T (serotype 5a). Primers were designed using the genome of 2457T to cover the entire GI with six overlapping PCR fragments (Fig. 2). The six PCR fragments were amplified from all but one of the 60 isolates; in this last strain, only PCR fragment 6 that does not contain any *fim* gene was amplified. The size of PCR fragment 2 containing *fimD* was 4.2 kb in 39 strains and 3.4 kb in 20 strains (Table 2); these sizes are consistent with the presence of IS*1* in *fimD* in 39 strains (as in 2457T and Sf301) and its absence in the remaining 20 strains (as in Sf8401). Accordingly, *fimD* is inactivated by IS*1* in most strains of serotype 2a (39 out of 43 strains), but not in strains of serotypes 1b (1 strain), 2b (7 strains), 3a (7 strains) and 3b (1 strain) (Table 2). These results were confirmed by nested PCR using internal primers for IS*1* (data not shown). Sizes of PCR fragments amplified from clinical strains indicated that none of these strains carried an IS in any other *fim* gene. We also analyzed 60 *S*. *sonnei* clinical strains; none of the six PCR fragments was amplified from any of these strains, indicating that, as in the sequenced genome of Ss046, the *fim* cluster is not present in these *S*. *sonnei* Chilean clinical isolates.

**Table 2:** Distribution of inactivation events of the *fim* cluster among *S*. *flexneri* Chilean clinical isolates clinical and reference strains.

**Serotype**	**Nature of strains** ^a^	**Number of strains**	**IS*1* in *fimD***	**IS*1* in *fimI***	**Non sense mutation in *fimB*** ^b^
**1b**	clin	1	0	0	1
**2a**	ref	2	2	0	2
**2a**	clin	44	39	0	43^c^
**2b**	clin	7	0	0	7
**3a**	clin	7	0	0	7
**3b**	clin	1	0	0	1
**5a**	ref	1	0	1	1
**5b**	ref	1	0	1	1
**Total**		64	41	2	63^c^

^a^ Reference strains (ref) are 2457T and Sf301 (serotype 2a), M90T (serotype 5a) and Sf8401 (serotype 5b). Clinical strains (clin) were isolated from patients in Chile during the period 2004–2006.

^b^ For clinical isolates, the nonsense mutation at codon 162 of *fimB* was tested by restriction analysis of PCR fragment 3 using *Bfa*I.

^c^ PCR fragment 3 was not amplified from one of the 44 strains tested.

In the sequenced *S*. *flexneri* genomes, the mutation introducing a nonsense codon at codon 162 of *fimB* creates a *Bfa*I restriction site (ATC *T*AG). To determine whether *S*. *flexneri* Chilean clinical isolates carry the same mutation in *fimB*, PCR fragment 3 containing *fimB* was digested with *Bfa*I; sizes of *Bfa*I fragments were consistent with the presence of a *Bfa*I site at codon 162 of *fimB* in all strains (Table 2). Even though the *fim* cluster is present in almost all *S*. *flexneri* Chilean clinical isolates and is presumably intact (*i*.*e*. not interrupted by any IS) in 20 strains, *fimB* is inactivated by the same mutation in all strains (Table 2). Restriction analysis of the fragment encompassing *fimS* amplified from ten clinical isolates that did not contain an IS in any *fim* genes indicated that the promoter of the *fim* operon is in the OFF orientation in all these isolates (data not shown), as it is in all the 129 *Shigella* genome sequences and WGS results available.

To determine whether the nonsense mutation in *fimB* is present in all *S*. *flexneri* strains, we analyzed results of two WGS projects, one encompassing 59 strains covering 14 serotypes (1a, 1b, 1d, 2a, 2b, 3a, 3b, 4a, 4av, 4b, X, Xv, Y and Yv) isolated from different provinces of China between 1997 and 2006 [40] and the other encompassing the 16 type strains covering all the established serotypes (1a, 1b, 1c, 2a, 2b, 3a, 3b, 3c, 4a, 4b, 5a, 5b, 6, X, Y and E1037) isolated from various regions between 1949 and 1972 and held at Public Health England (PHE) [41], as well as the sequence of the *S*. *flexneri* 2a strain NCTC1 isolated from a English soldier on the Western front in 1915 [37]. In all these strains, but the serotype 6 strain of the PHE collection, *fimB* carries the same mutation in codon 162. Further analysis of WGS results available for three other serotype 6 strains, CFSAN027317, CCH60 and CDC 796–83 confirmed that serotype 6 strains do not contain the mutation in *fimB* and, instead, all contain both a nonsense codon at codon 563 of *fimD* and an insertion of IS*629* at codon 264 of *fimH*. The same two mutations are also present in the *S*. *boydii* strains Sb227 and 3594–74 (both of serotype 4) and 4444–74 (serotype 2). These later observations are consistent with previous analysis showing that *S*. *flexneri* serotype 6 strains are more closely related to *S*. *boydii* strains than to any other *S*. *flexneri* strains [11], [46].

### Construction of a *S*. *flexneri* strain producing functional type 1 fimbriae

**Fig 3:**
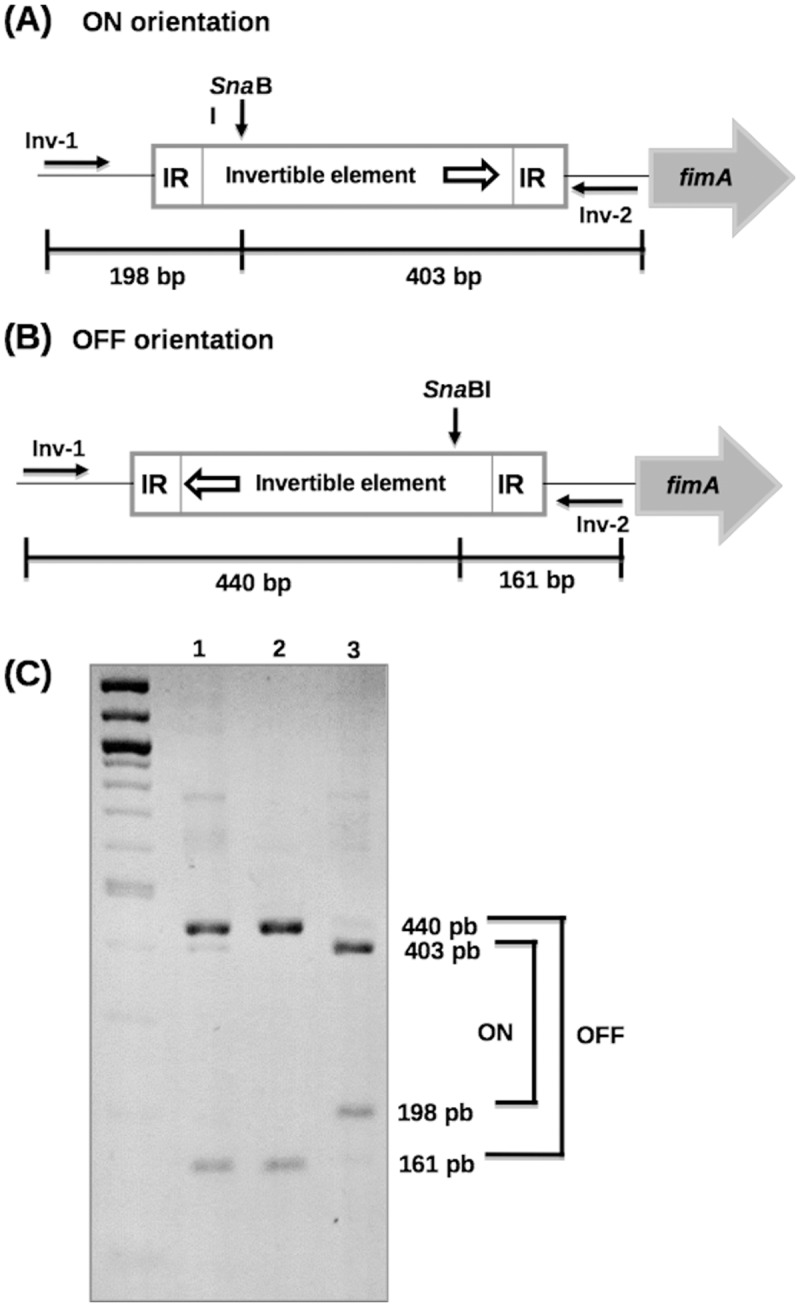
Determination of the orientation of the *fimS* invertible element. Schematic representations of the region encompassing *fimS* in the ON and OFF orientations are shown in panels A and B, respectively; positions of *fimS* (box), inverted repeats (IR) on both sides of *fimS*, the promoter of the *fim* operon (small open arrow), the 5' end of *fimA* (large grey arrow), primers Inv-1 and Inv-2 (small black arrows), the *Sna*BI cleavage site and sizes of the *Sna*BI digestion products of the PCR fragment amplified by using Inv-1 and Inv-2 are indicated for each orientation. (C) The region encompassing *fimS* was amplified by PCR by using primers Inv-1 and Inv-2 and digested with *Sna*BI and restriction fragments were resolved on a 2% agarose gel; lane 1, M90T/pSH2 after growth for 24 h in static conditions; lane 2, M90T/pACYC184 after growth for 24 h in static conditions; lane 3, M90T/pSH2 after growth for 48 h in static conditions. The *fimS* region is carried by both the chromosome and the plasmid pSH2 in M90T/pSH2 and only by the chromosome in M90T/pACYC184; due to the higher copy number of pSH2 as compared to the chromosome, *fimS* was preferentially amplified from the pSH2 plasmid in M90T/pSH2. Diagrams on panels A and B were adapted from [44].

To analyze a *S*. *flexneri* strain producing fimbriae, the *S*. *flexneri* strain M90T was transformed with the plasmid pSH2, a derivative of the vector pACYC184 carrying the entire *E*. *coli fim* cluster [22]. To enrich the proportion of bacteria in which the *fim* promoter was in the ON orientation, bacteria were first cultured in static conditions and the orientation of the promoter was monitored by restriction analysis of a PCR product covering *fimS* (Fig. 3). Results indicated that the *fim* promoter was in the ON orientation in ~80% of the plasmid population and RT-PCR on *fimA*, *fimH* and *fimD* confirmed the transcription of *fim* genes in M90T/pSH2 (data not shown). To visualize production of type 1 fimbriae at the surface of bacteria, electron microscopy images of negatively stained M90T bacteria harboring pSH2 or pACYC184 were obtained.

**Fig 4:**
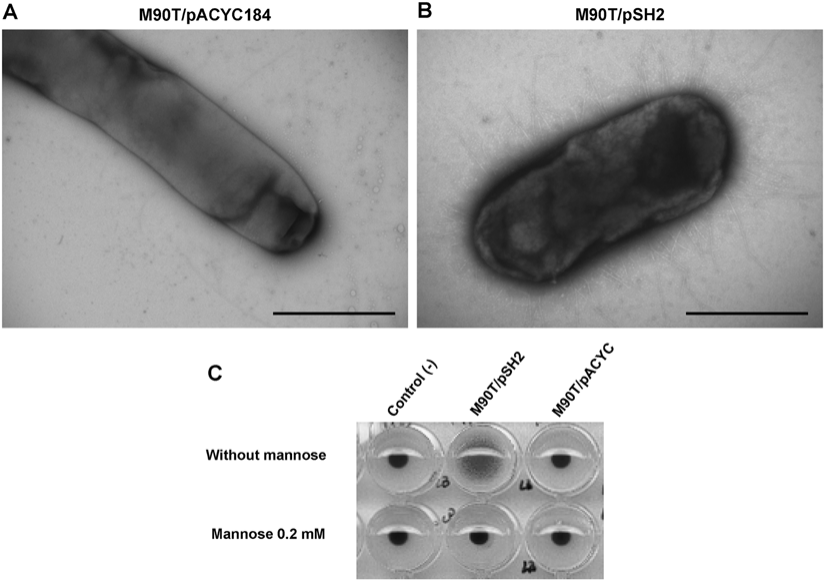
Characterization of type 1 fimbriae production in derivatives of the *S*. *flexneri* strain M90T. Representative electron microscopy pictures of derivatives of *S*. *flexneri* strain M90T harboring pACYC184 (vector) or pSH2 (carrying the *fim* operon) are shown in panels A and B, respectively. Scale bar, 1 μm. Results of an haemagglutination assays performed in the absence or in the presence of 0.2 mM mannose with the *S*. *flexneri* strain M90T harboring either pSH2 or pACYC184 are shown in panel C.

Electron microscopy analysis showed the presence of fimbriae at the surface of 74% of bacteria harboring pSH2, but not of bacteria harboring pACYC184 (Fig. 4). Bacteria producing type 1 fimbriae promote agglutination of erythrocytes in the absence, but not in the presence of mannose [43], [44]. Haemagglutination assays performed using Guinea pig erythrocytes confirmed that M90T/pSH2, but not M90T/pACYC184, expressed functional fimbriae (Fig. 4).

### Effect of type 1 fimbriae production on the interaction of *S*. *flexneri* with epithelial cells

**Fig 5:**
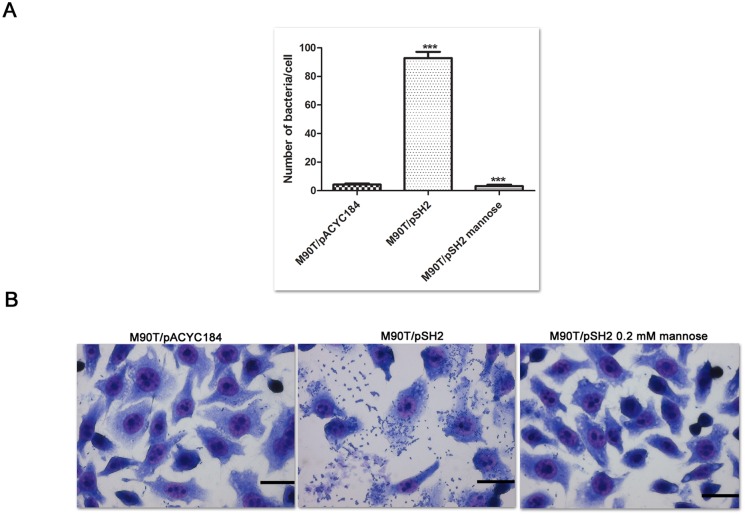
Adhesion of S. *flexneri* bacteria expressing type 1 fimbriae to HeLa cells. HeLa cells infected for 30 min with M90T/pACYC184 or M90T/pSH2 in the absence or in the presence of 0.2 mM mannose were fixed with ethanol and stained with Giemsa. Numbers of bacteria per cell are shown in (A). Data are the means of three independent experiments performed in duplicate; *** denotes P-value < 0.001. Representative pictures of Giemsa stained cells are shown in (B); scale bar, 40 μm.

To investigate the potential consequence of type 1 fimbriae production on the interaction of *Shigella* with epithelial cells, we monitored adhesion of M90T/pSH2 to HeLa cells. Following centrifugation of bacteria onto semi-confluent cell monolayers, infected cells were incubated for 30 min at 37°C, washed, fixed with ethanol and stained with Giemsa. Observation of infected cells revealed a 50-fold increase in the number of cell-associated M90T/pSH2 as compared to M90T/pACYC184 (Fig. 5). The increased adhesion of M90T/pSH2 was abolished in the presence of mannose, confirming that it was dependent upon functional type 1 fimbriae (Fig. 5).

**Fig 6:**
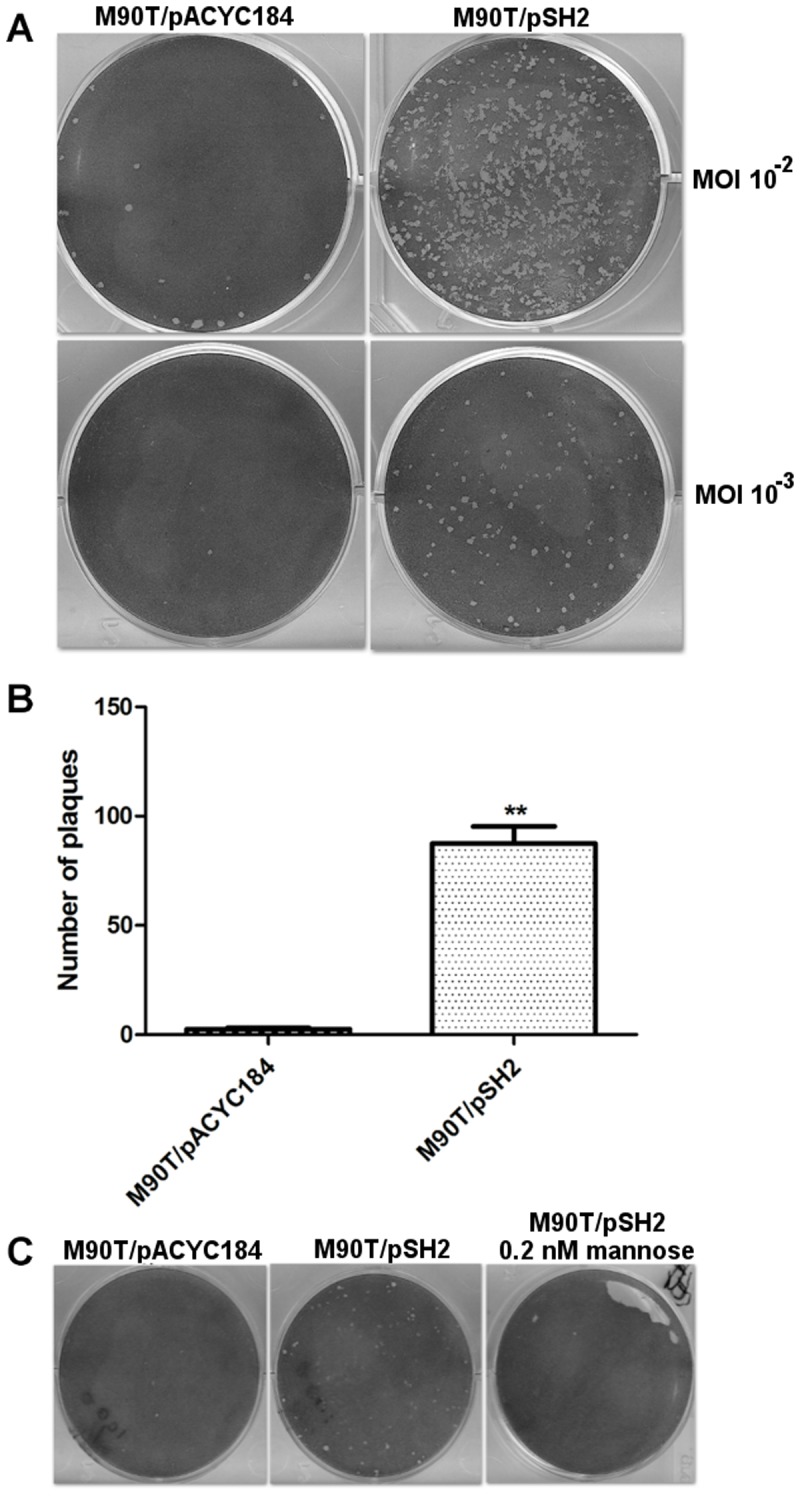
Invasion and dissemination of *S*. *flexneri* bacteria expressing type 1 fimbriae. (A) Confluent monolayers of TC7 cells were infected with M90T/pACYC184 or M90/pSH2 at multiplicities of infection (MOI) of 0.01 (upper row) or 0.001 (lower row), overlaid with gentamicin-containing agar, incubated at 37°C for 72 h, fixed and stained with Giemsa. (B) The graph indicates the number of plaques formed in the monolayer infected with either M90T harboring pACYC184 (vector) or pSH2 (carrying the *fim* operon), as calculated using different MOIs. Values are the means and SD of three independent experiments, each performed in triplicate at a MOI of 0.001; ** indicates P-value between 0.001 and 0.01. (C) Confluent monolayers of TC7 cells were infected with M90T/pACYC184 or M90/pSH2 at a MOI of 0.001 in the absence or in the presence of 0.2 mM mannose during the first 2 h (entry step).

To evaluate invasion and dissemination of bacteria in a confluent monolayer of TC7 cells, we used the plaque assay that does not involve any centrifugation of bacteria onto the cells; in this assay, the number of plaques is indicative of the ability of bacteria to enter epithelial cells and the size of plaques is indicative of the ability of bacteria to spread from cell to cell [45]. M90T/pSH2 induced the formation of approximately 50 times more plaques than M90T/pACYC184, an increase that was abolished when invasion was performed in the presence of mannose (Fig. 6). Plaques of similar sizes were produced by the two strains, suggesting that expression of fimbriae on the bacterial surface had no effect on the capacity of bacteria to disseminate from cell to cell. These results demonstrated the increased invasive capacity conferred upon *Shigella* by production of fimbriae.

## Discussion

This study analyzed the presence, integrity and functionality of the *fim* cluster carried by the GI inserted next to *leuX* in the genome of members of *Shigella* spp. and EIEC. The presence of all or part of the *fim*, *yjfATS* and *fec* clusters in these strains suggests that the *leuX* GI initially carried these three gene clusters. Analysis of published genome sequences indicated that the *fim* cluster is either deleted or inactivated in members of all *Shigella* and EIEC phylogenetic groups: (i) in group A, there is a deletion encompassing *fimBEAICDF* (in EIEC 53638); (ii) in group S1, there is a mutation introducing a nonsense codon at codon 563 of *fimD*, an insertion of IS*629* after codon 264 of *fimH* and either a frameshift mutation at codon 25 of *fimD* (in *S*. *boydii* CDC3083-94) or a deletion of *fimEBA* (in *S*. *boydii* Sb227); (iii) in group S2, the *fim*, *yjhATS* and *fec* clusters and the *uxuABR* and *gntP* neighboring genes are absent in *S*. *dysenteriae* 155–74 and only the 3' part of *fimD* and the *fimFGH* genes are present in *S*. *boydii* strains CIP52-54 and CIP56-18; (iv) in group S3, there is a mutation introducing a nonsense codon at codon 162 of *fimB* in all *S*. *flexneri* strains except strains of serotype 6, as well as an insertion of IS*1* in *fimD* in some serotype 2a strains and in *fimI* in some serotype 5b strains; (v) in group SD1, there is a deletion encompassing the whole *fim* cluster and the adjacent *uxuABR* and *gntP* genes (in *S*. *dysenteriae* Sd197); (vi) in group SS, there is a deletion of the whole *fim* cluster in *S*. *sonnei* Ss046, all 60 Chilean clinical isolates tested and most strains for which WGS data are available and only remnants of the *fim* cluster are present in the *S*. *sonnei* strain 53G and a few other strains. PCR and restriction analysis of *fimB* from 60 *S*. *flexneri* Chilean clinical isolates and the survey of the genome sequences and WGS results of over 80 *S*. *flexneri* strains from various origins [37], [40]- [41] indicated that the very same mutation in *fimB* is present in all *S*. *flexneri* strains, except strains of serotype 6. The four serotype 6 strains we analyzed contain both the same mutation in *fimD* and the same insertion of IS*629* in *fimH*; the very same two mutations are present in some *S*. *boydii* strains, consistent with the close relationship between *S*. *flexneri* serotype 6 and *S*. *boydii* strains [11], [46]. The presence of the same mutation in *fimB* in *S*. *flexneri* strains of all serotypes (except strains of serotype 6 discussed above) isolated over a period of almost a century from three continents, *i*.*e*. in 1915 in France (NCTC1), in 1954 in Tokyo (2457T), in 1955 in Mexico City (M90T), in 1984 in Beijing (Sf301), from 1949 to 1972 in various places (PHE collection), in the early 2000s in Santiago (clinical isolates used in this study) and from 1997 to 2006 in various provinces of China, suggests that this mutation occurred prior to serotype diversification [11]. Even though the nature of the initial mutation that led to inactivation of a gene cluster cannot be demonstrated in the case of deletions encompassing several genes, there is evidence that inactivation of the *fim* cluster is due to different point mutations in at least three cases.

The identification of mutations inactivating the *fim* cluster in *Shigella* spp. shed light on previous observations by Snellings *et al*. reporting that bacteria expressing type 1 fimbriae were recovered from a subset of *Shigella* strains, albeit at a low frequency, after five to ten serial transfers in static culture conditions and that fimbriae expression was accompanied by inversion of *fimS* in the ON orientation in *S*. *flexneri* Fim+ derivatives [47]. It is likely that *fimB* was inactivated in the *S*. *flexneri* strains used by Snellings *et al*., as it is in all *S*. *flexneri* genomes and strains examined in the present study; accordingly, the reported low frequency of switching to the fimbriated phase was probably due to the necessity of reverting the non-sense mutation in *fimB* (or expressing a suppressor tRNA) prior to obtaining inversion of *fimS* and expression of the *fim* operon. No fimbriated derivatives were obtained from M90T [47] in which, as shown here, *fimI* is also inactivated by an IS*1*. Recovery of fimbriated derivatives from one *S*. *boydii* strain and one *S*. *dysenteriae* strain, the serotypes of which were not reported, is more difficult to explain from the analysis of *S*. *boydii* and *S*. *dysenteriae* available genome sequences. Indeed, there is both a nonsense mutation at codon 564 of *fimD* and an insertion of IS*629* at codon 264 of *fimH* in *S*. *boydii* strains Sb27 (serotype 4), CDC3083-94 (serotype 18), 4444–74 (serotype 2) and 3594–74 (serotype 4); *fimBEAIC* and the 5' part of *fimD* are missing in *S*. *boydii* strains CIP52-54 and CIP56-18 (serotypes 7 and 11) and the whole *fim* cluster is absent from the nine other *S*. *boydii* strains for which WGS results are available in GenBank. The *fim* cluster is absent from the complete genome of the *S*. *dysenteriae* strain Sd197, as well as from WGS results of seven other *S*. *dysenteriae* strains, and only a truncated *fim* cluster interrupted by 3 IS is present in WGS results of the *S*. *dysenteriae* strain 222–75. Future analysis of larger datasets for *S*. *boydii* and *S*. *dysenteriae* genomes will help to define further inactivation events of the *fim* cluster in these *Shigella* spp.

To investigate the potential role of fimbriae production on the interaction of *Shigella* with host cells, we used a *S*. *flexneri* strain expressing the *fim* operon from *E*. *coli*. Production of fimbriae increased the ability of bacteria to adhere to epithelial cells *in vitro*, consistent with the reported role of type 1 fimbriae in promoting adhesion of *E*. *coli* to epithelial cells [48], [49]. Increasing the capacity of *S*. *flexneri* to adhere to epithelial cells by experimental expression of the adhesin AfaE or coating bacteria with poly-lysine was reported to increase entry of bacteria into cells *in vitro* [50], [51]. However, it was conceivable that exposure of long fimbriae on the surface of bacteria might interfere with either the delivery of T3S effectors or the accessibility of IcsA that are involved in entry and intracellular mobility of bacteria, respectively. In the plaque assay, in which the interaction between bacteria and cells is not forced by a centrifugation step, the invasive capacity of the fimbriated strain was 50 folds higher than that of the non-fimbriated strain. Plaques of similar size were produced by the Fim+ and Fim- strains, indicating that production of fimbriae did not affect the ability of *S*. *flexneri* to disseminate from cell to cell. These results indicated that production of fimbriae does not interfere with delivery of T3S effectors and, by increasing the ability of bacteria to interact with epithelial cells, increases the ability of these bacteria to invade these cells *in vitro*.

In deciphering the interactions pathogenic bacteria establish with their host(s), much attention has been paid to the identification of their specific virulence determinants, most often acquired by lateral transfer. However, both the loss and the gain of genetic material have contributed to the adaptation of bacteria to new environments. The importance of pathoadaptive mutations is increasingly recognized [16], [52], [53]. The presence of antigenic surface structures that do not have an overall favorable role in infection are counter-selected, possibly because they increase detection of bacteria by the innate and adaptive immune systems. Indeed, genes encoding the flagellum and curli are inactivated in *Shigella* spp. [54]–[56]. Moreover, expression of other immunogenic surface structures, such as the LPS, is modified at different stages of infection; in an initial phase of infection of epithelial cells, lipid A acylation is decreased, likely allowing *Shigella* to evade the immune detection [57]. Even though expression of type 1 fimbriae in *Shigella* produced a hyper-invasive phenotype *in vitro*, it may be detrimental to bacterial survival in the gut, given that expression of a highly immunogenic structure at the onset of infection might trigger a strong immune response. Moreover, it was shown that a fimbriated *Shigella* strain was more sensitive to killing by human granulocytes than a non-fimbriated strain [58] and that expression of FimH in uropathogenic *E*. *coli* (UPEC) induced rapid neutrophil recruitment in mice [59]. There is accumulating evidence indicating that *Shigella* inhibits the inflammatory response, including during invasion of epithelial cells [4], [60]–[69]. For instance, the T3S effector IpgD was shown to prevent the hemichannel-dependent secretion of the endogenous danger signal ATP, an early alert response to infection in intestinal epithelial cells [69]. The finding that *Shigella* actively dampens the immune response highlights the necessity of these invasive bacteria to avoid immune detection in the first place. The observation that independent mutations led to inactivation of fimbriae production in spite of the increased invasion capacity conferred upon *Shigella* by fimbriae expression suggests that these mutations might correspond to pathoadaptive events.
